# Supplementary material for: Crystal structure of the INTS3/INTS6 complex reveals the functional importance of INTS3 dimerization in DSB repair
Source: Cell Discov. 2021 Aug 17;7:66. doi: 10.1038/s41421-021-00283-0 (PMC8368002; doi:10.1038/s41421-021-00283-0)
Supplement: Supplementary file 1 — Supplementary Information [file 41421_2021_283_MOESM1_ESM.pdf]

Supplementary Data

Crystal Structure of INTS3/INTS6 complex reveals Functional Importance of INTS3 dimerization in DSB Repair

Yu Jia[1, #], Zixiu Cheng[1, #], Sakshibeedu R Bharath[2, #], Qiangzu Sun[1], Nannan Su[1], Jun Huang[1, *] and Haiwei Song[1,2,3, *]

Supplementary Table S1 Data collection and refinement statistics

	Native	Semet
**Data collection**		
Space group	$P2_1$	$P2_1$
Cell dimensions		
a, *b*, *c* (Å)	60.57, 126.03, 84.44	60.87, 125.93, 84.78
β (°)	102.12	102.14
		Peak
Wavelength	1.000	0.966
Resolution (Å)	49.45 - 2.40 (2.49 - 2.40)	43.25 - 2.60 (2.72 - 2.60)
R_{merge}	0.08 (0.75)	0.09 (0.52)
$I / \sigma I$	4.9 (1.3)	8.5 (2.2)
CC1/2	0.995 (0.53)	0.993 (0.61)
Completeness (%)	90.0 (92.9)	99.3 (99.6)
Redundancy	2.7 (2.7)	3.2 (3.2)
**Refinement**		
Resolution (Å)	43.95 - 2.40 (2.49 -2.40)	
No. reflections	43428 (2124)	
R_{work} / R_{free}	0.20 / 0.25	
No. atoms		
Protein	6005	
Water	257	
B-factors		
Protein	53.7	
Water	49.9	
R.m.s deviations		
Bond lengths (Å)	0.003	
Bond angles (°)	0.588	
Ramachandran plot (% residues)		
Allowed	96.0	
Generously allowed	4.0	
Disallowed	0.0	

Values in parentheses are for the highest-resolution shell.

Supplementary Figures

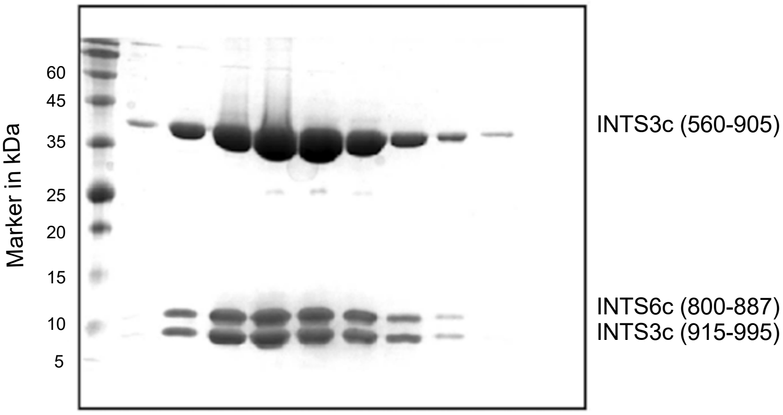

Figure S1. SDS-PAGE of the crystallized INTS3c/INTS6c complex

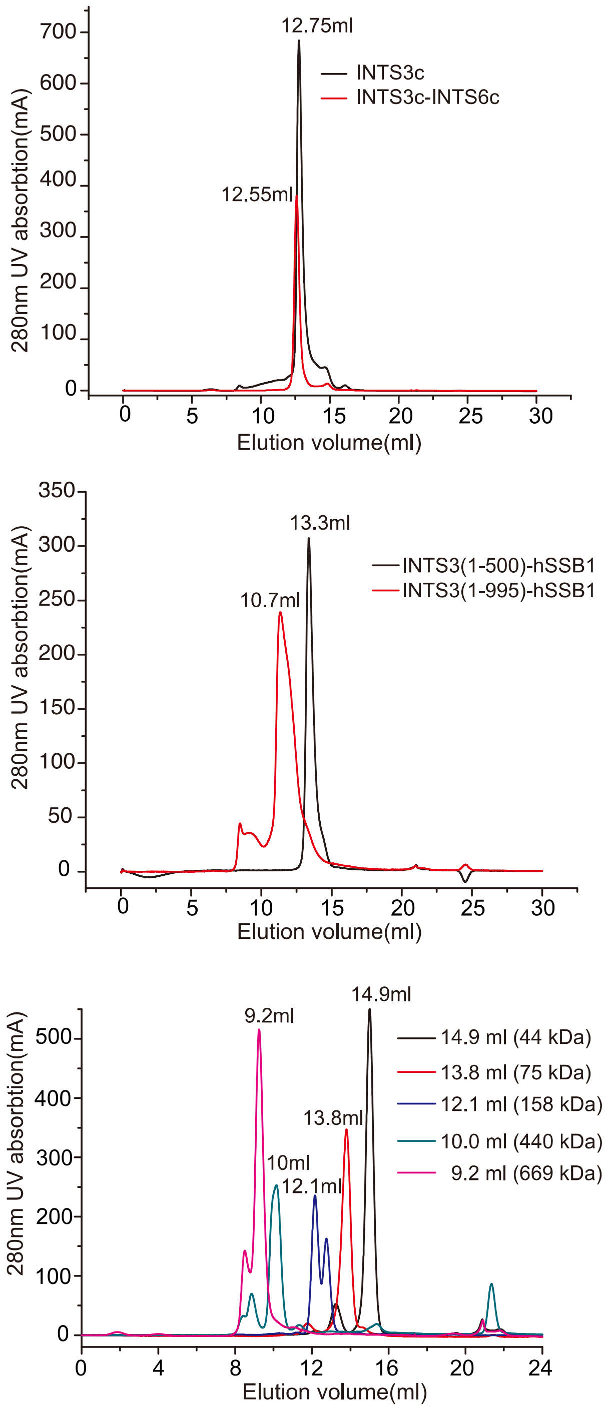

Figure S2. Gel filtration profiles of INTS3c, INTS3c/INTS6c, INTS3(1-500)/hSSB1 and INTS3(1-995)/hSSB1

Purified INTS3c, INTS3c/INTS6c, INTS3(1-500)/hSSB1 and INTS3(1-995)/hSSB1 elute at 12.5, 12.7, 13.3 and 10.7 ml, respectively from S200 superdex gel filtration column (top and middle panels). Comparison with elution volumes of the molecular weight markers in the same column (bottom panel) suggests that protein complexes, INTS3c, INTS3c/INTS6c and INTS3 (1-995)/hSSB1 are likely to be dimers in solution and INTS3(1-500)/hSSB1 is a monomeric complex.

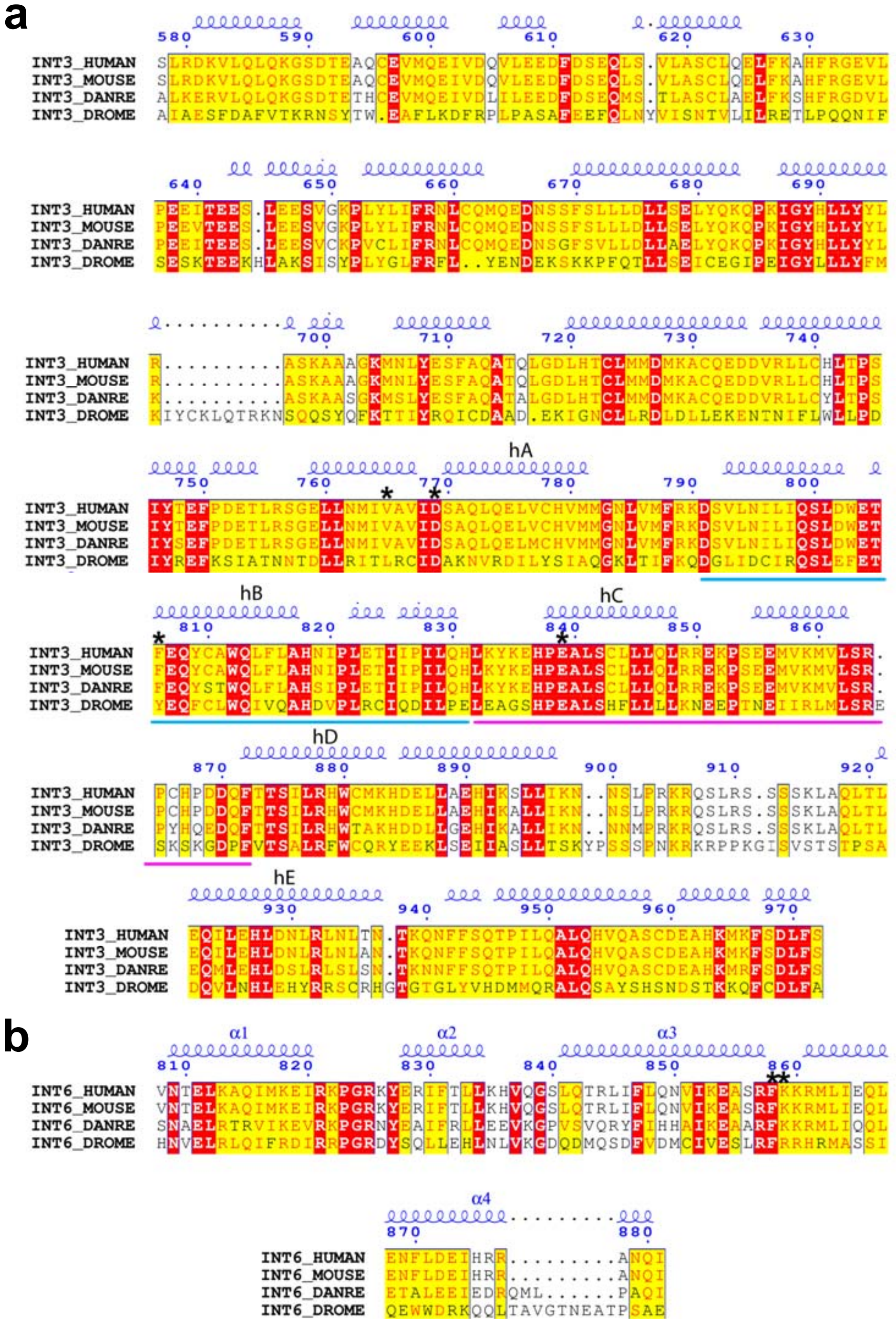

Figure S3. Sequence alignments of INTS3c and INTS6c with homologs from human, mouse, zebra fish and Drosophila

(**a**)Sequence alignment of human (HUMAN), mouse (MOUSE), zebrafish (DANRE) and Drosophila (DROME) INTS3c reveal conserved features. The secondary structures of human INTS3c are shown on top of the alignment. The helices involved in INTS3c dimer formation are labeled hA, hB, hC, hD and hE. **(b)** Sequence alignment of human (HUMAN), mouse (MOUSE), zebrafish (DANRE) and Drosophila (DROME) INTS6c with secondary structures of human INTS6c shown on top of the alignment. The residues mutated in the study, Val767, Asp769, Phe806, Glu839 in INTS3c and Phe858 and Lys859 in INTS6c are marked with asterisk on top of the alignment. The two segments in deletion constructs of INTS3, Δ791-831 and Δ832-872 are marked as blue and pink bars, respectively underneath the alignment.

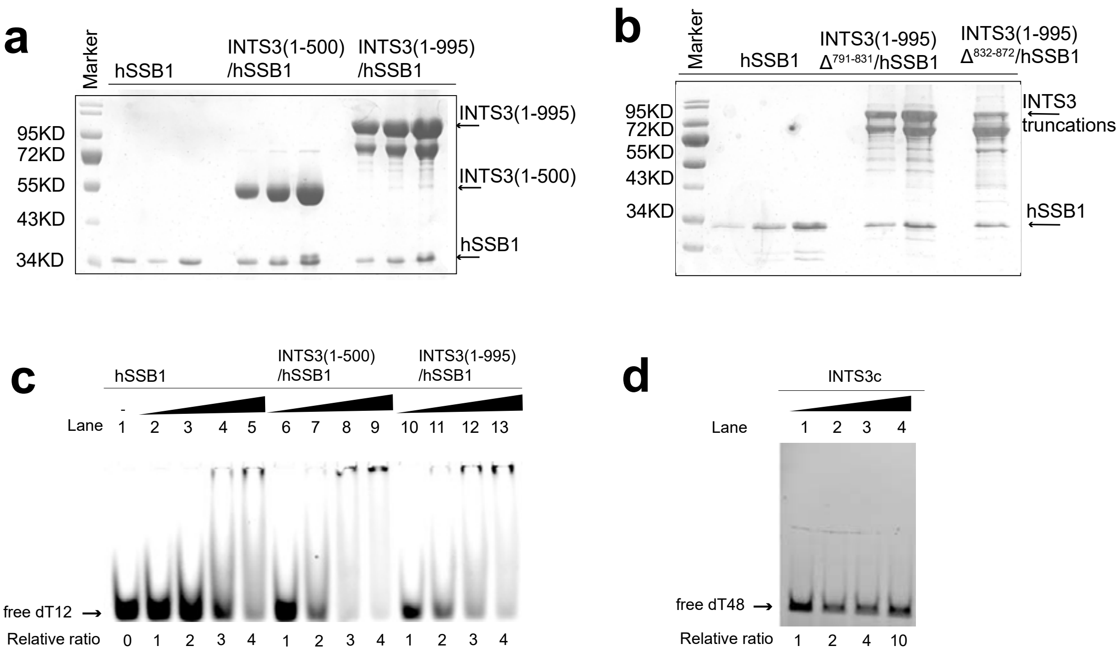

Figure S4. Purification of various INTS3 complexes and their interaction with ssDNA

(a) Coomassie blue stained SDS-PAGE shows hSSB1, INTS3$_N$/hSSB1 and INTS3 (1-995)/hSSB1. Arrows point to protein bands. **(b)** Coomassie blue stained SDS-PAGE shows hSSB1, INTS3 (1-995)$^{\Delta 791-831}$/hSSB1 and INTS3 (1-995)$^{\Delta 832-872}$/hSSB1. Arrows point to protein bands. **(c)** Interaction of dT12 with hSSB1, INTS3$_N$/hSSB1 and INTS3 (1-995)/hSSB1 as examined by electrophoretic mobility assay (EMSA). **(d)** Interaction of dT48 with INTS3c as examined by electrophoretic mobility assay (EMSA).

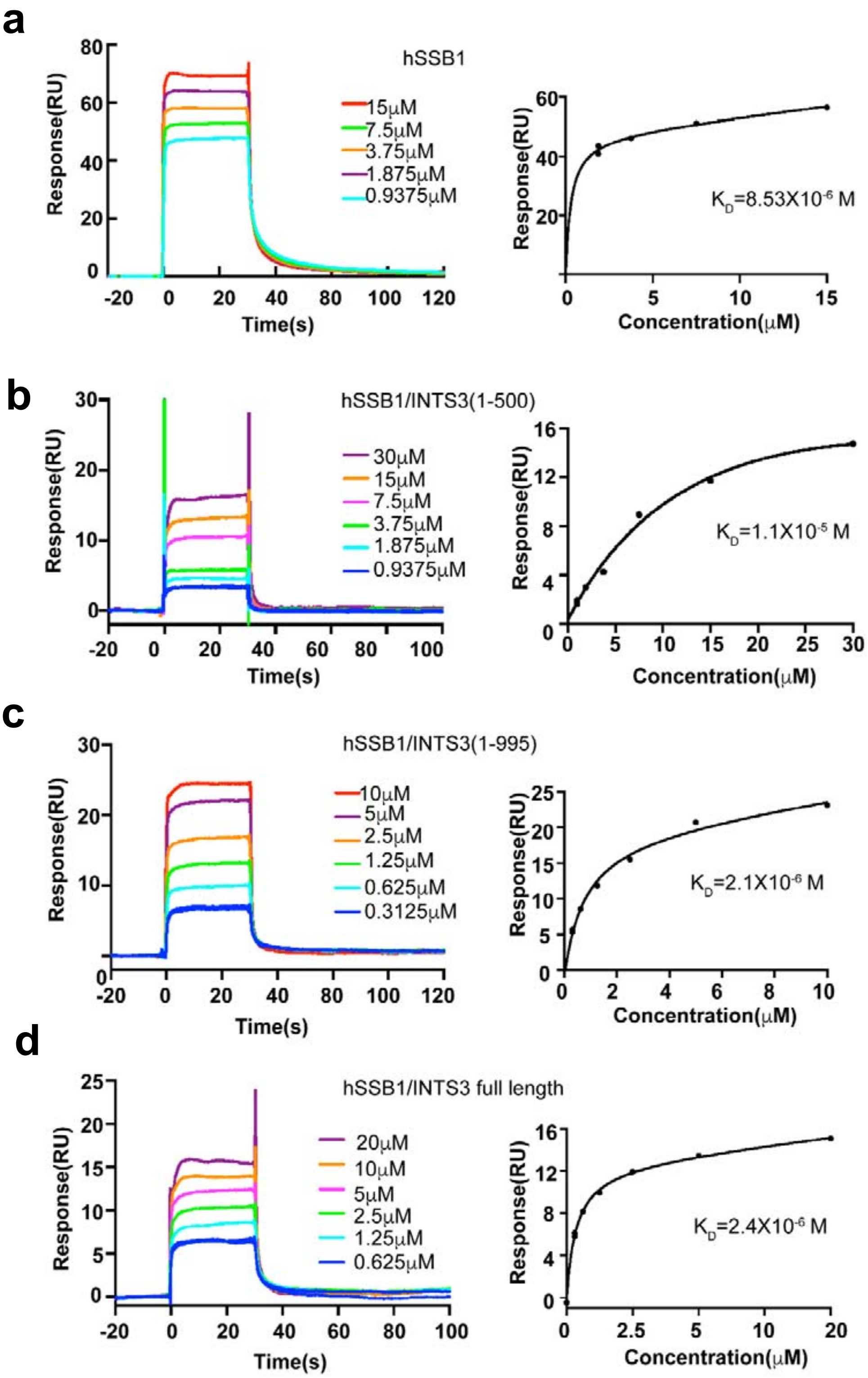

Figure S5. Interaction of hSSB1, INTS3/hSSB1 and its mutants with ssDNA

Sensograms resulting from Surface Plasmon resonance (SPR) assay for interaction between (**a**) immobilized hSSB1 and dT48 (**b**) immobilized INTS3(1-500) and dT48 (**c**) immobilized INTS3 (1-995)/hSSB1 and dT48 and (**d**) immobilized full length INTS3/hSSB1 and dT48. Dissociation constant (K_D) was calculated by fitting to a one-site binding model.
